# Personal Network Bridging Potential Among Rural and Older Populations

**DOI:** 10.1093/geroni/igab046.988

**Published:** 2021-12-17

**Authors:** Adam Roth, Siyun Peng, Brea Perry

**Affiliations:** Indiana University, Bloomington, Indiana, United States

## Abstract

Personal social networks play a fundamental role in the daily lives of older adults. Although many studies examine how life course factors and personal preferences shape network formation, fewer consider how the places in which older adults live present opportunities and obstacles to cultivate social relationships. In the present study, we explore how geographic context is associated with the ability to interact with non-overlapping social groups within one’s personal network (i.e., network bridging). This unique network formation offers older adults access to diverse social stimuli, non-redundant information, and social autonomy. By analyzing data from the Person-to-Person Health Interview Survey (N=709), we found that a minority of respondents reported the ability to bridge social groups within their networks. Respondents residing in rural and semi-rural counties engaged in fewer non-overlapping social groups compared to those residing in urban counties. These findings suggest that the communities in which older adults live condition opportunities for accessing unique social resources. Identifying the link between geographic residence and personal network structure has important implications for how individuals navigate the uncertainty and elevated support needs of later life. Additional research adopting a social network perspective is needed to provide insight into geographic health disparities occurring among the older population.

